# Synthesis, crystal structure and Hirshfeld surface analysis of aqua­(3-meth­oxy­cinnamato-κ*O*)bis­(1,10-phenanthroline-κ^2^ *N*,*N*′)cobalt(II) nitrate

**DOI:** 10.1107/S2056989022009781

**Published:** 2022-10-11

**Authors:** Asma Lehleh, Mehdi Boutebdja, Chahrazed Beghidja, Adel Beghidja

**Affiliations:** a Unité de Recherche de Chimie de l’Environnement et Moléculaire Structurale (CHEMS), Université Frères Mentouri Constantine 1, 25017, Constantine, Algeria; b Laboratoire Technologie des Matériaux Avancés, École Nationale Polytechnique de Constantine, Nouvelle Ville Universitaire Ali Mendjeli, 25000, Constantine, Algeria; Texas A & M University, USA

**Keywords:** 3-meth­oxy­cinnamic acid, X-ray diffraction, crystal structure, Hirshfeld surface, N-donor

## Abstract

The title compound, [Co(C_10_H_9_O_3_)(C_12_H_8_N_2_)_2_(H_2_O)]NO_3_ (I), crystallizes in the triclinic space group *P*  with a monomeric [Co(3-meo-cin)(phen)_2_(H_2_O)]^+^ cation and a nitrate anion (3-meo-cin = 3-meth­oxy cinnamic acid) in the asymmetric unit. The Co^II^ ion is coordinated by four N atoms from two 1,10-phenanthroline ligands and two O atoms, the first from a meth­oxy cinnamate ligand and the second from a coordinated water mol­ecule, forming a distorted octa­hedral geometry. Discrete entities of the cation and nitrate anion are formed by water–nitrate O—H⋯O and phen–nitrate C—H⋯O hydrogen bonds. The components are further assembled into chains along the *c-*axis direction. Layers are than formed by slipped π–π stacking inter­actions parallel to the *bc* plane. The inter­molecular inter­actions in the crystal structure were qu­anti­fied and analysed using Hirshfeld surface analysis.

## Abstract 2

The title compound crystallizes in the triclinic space group *P* 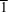 with a monomeric [Co(3-meo-cin)(phen)_2_(H_2_O)]^+^ cation and a nitrate anion (3-meo-cin = 3-meth­oxy cinnamic acid) in the asymmetric unit. The Co^II^ ion is coordinated by four N atoms from two 1,10-phenanthroline ligands and two O atoms, the first from a meth­oxy cinnamate ligand and the second from a coordinated water mol­ecule, forming a distorted octa­hedral geometry.

## 1. Chemical context

Cinnamic acid (3-phenyl-2-propenoic acid), a derivative of phenyl alanine, comprises a relatively large family of organic isomers (Ferenc *et al.*, 2012 ▸; Madhurambal *et al.*, 2010 ▸). Cinnamic acid and its derivatives exhibit biological activities (Rychlicka *et al.*, 2021 ▸) including anti­bacterial (Sova, 2012 ▸), anti­fungal (Ruwizhi & Aderibigbe, 2020 ▸) and anti­parasitic properties (Kanaani & Ginsburg, 1992 ▸) as well as a variety of pharmacological properties (Adisakwattana *et al.*, 2008 ▸) including hepatoprotective (Lee *et al.*, 2002 ▸), anti­malarial (Wiesner *et al.*, 2001 ▸), anti­oxidant (Natella *et al.*, 1999 ▸), anti­tumoral (Ferenc *et al.*, 2012 ▸), anti­hyperglycemic and anti­tyrosinase activities (Lee, 2002 ▸). Cinnamic acid and related compounds have attracted particular attention over the last few decades, not only for their biological activities, but also for their carboxyl­ate group. The popularity of such aromatic carb­oxy­lic acids as building blocks for generating metal–organic architectures can be explained by their coordination versatility and ability to act as multiple linkers (Lehleh *et al.*, 2015 ▸; Gu *et al.*, 2020 ▸), high thermal stability, tuneable deprotonation of –COOH groups, remarkable physicochemical properties, as well as the ability to function as hydrogen-bond donors and acceptors, thus facilitating the formation of intricate hydrogen-bonded networks (Gu *et al.*, 2020 ▸; Zhang *et al.*, 2019 ▸; Zhou *et al.*, 2019 ▸). Furthermore, bipyridyl-like ligands such as 2,2′-bi­pyridine and 1,10-phenanthroline used as auxiliary ligands, are usually used in the formation of different complexes with a variety of transition metals, because of their versatile roles such as in analytical chemistry, in catalysis, in electrochemistry, in ring-opening metathesis polymerization and biochemistry (Lehleh *et al.*, 2011 ▸). Additionally, the pyridine rings can not only inter­act with each other *via* π–π stacking inter­actions, but also act as hydrogen-bond donors and acceptors (Cao *et al.*, 2014 ▸; Hao *et al.*, 2011 ▸; Lehleh *et al.*, 2011 ▸). 
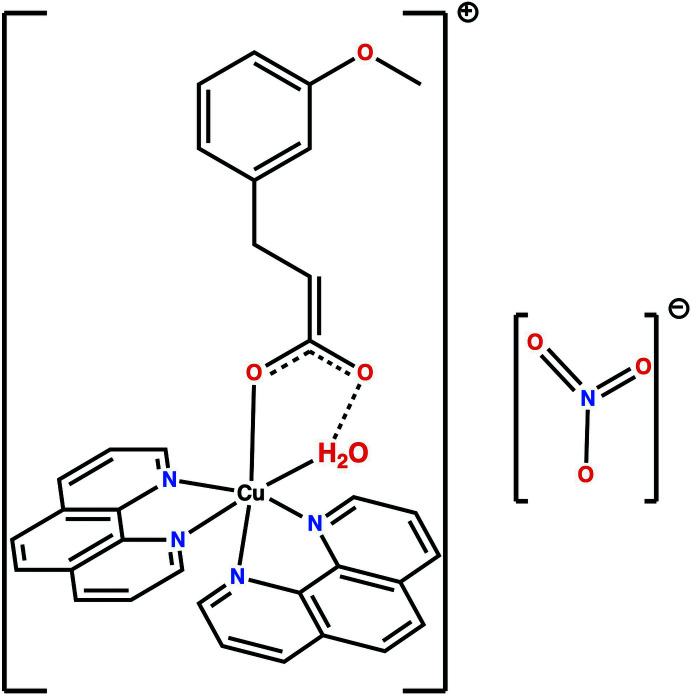


In this context, we report the synthesis, structural characterization and Hirshfeld surface analysis of the title compound [Co(C_10_H_9_O_3_)(C_12_H_8_N_2_)_2_(H_2_O)] NO_3_.

## 2. Structural commentary

**Figure 1:**
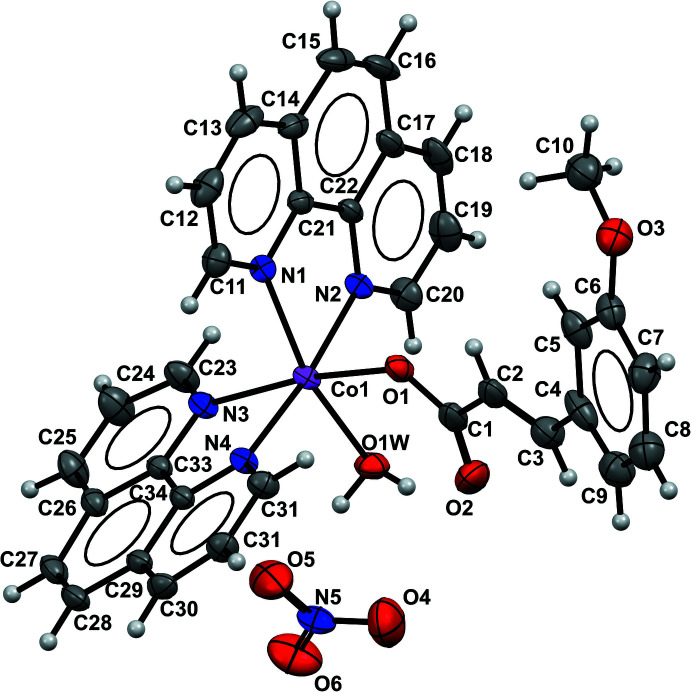
An ellipsoid plot of the title compound showing the atom-labelling scheme with ellipsoids drawn at the 50% probability level and H atoms shown as small spheres of arbitrary radii.

The asymmetric unit of the title compound, illustrated in Fig. 1 ▸, consists of a Co^II^ complex cation and one nitrate anion. The Co^II^ ion is in a distorted octa­hedral geometry, coordinated by two 1,10-phenanthroline (phen) units through both N atoms in the usual bidentate manner, one water mol­ecule and one 3-meth­oxy cinnamate in a monodentate fashion. The Co—N_phen_ bond distances range from 2.1356 (16) to 2.1488 (17) Å, while the Co—O_cin_ and Co—O_water_ bond lengths are 2.0525 (13) and 2.1011 (17) Å, respectively. The axial bond angles around the Co^II^ ions are in the range 166.30 (7)–173.94 (6)° (Table 1 ▸). The large deviation of the axial bond angles from an ideal octa­hedral geometry (180°) clearly indicates that the coordination environment around the Co^II^ ion is best described as distorted octa­hedral. The 3-meth­oxy cinnamate mol­ecule shows disorder over two positions with occupancies of 0.735 (6) and 0.265 (6).

## 3. Supra­molecular features

**Figure 2:**
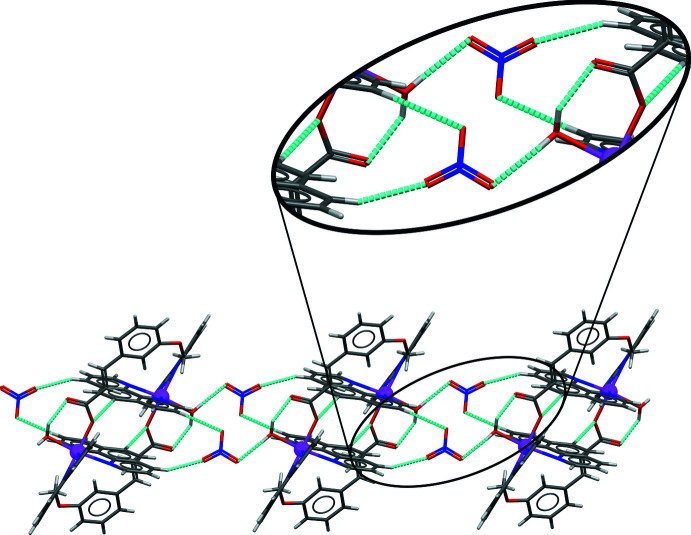
Crystal packing of the title compound shown in projection down the *c* axis illustrating chain formation along the *c-*axis direction by C—H⋯O hydrogen bonding (shown as dashed cyan lines).

**Figure 3:**
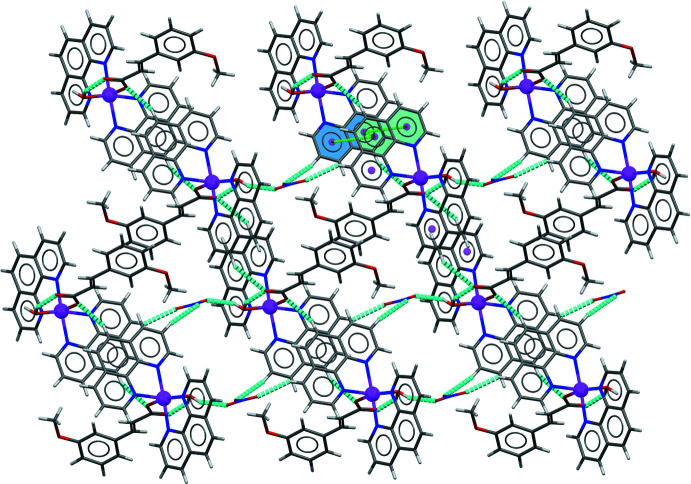
Crystal packing of the title compound showing the layers parallel to the *bc* plane formed by the π–π stacking inter­actions between the pyridyl rings of the 1,10-phenanthroline units (blue and cyan). Hydrogen bonds are shown by dashed cyan lines.

The structure presents extensive hydrogen bonding with numerical details given in Table 2 ▸. The coordinated water mol­ecule (O1*W*) forms hydrogen bonds with the non-coord­in­ating O atom of the carboxyl­ate group of the 3-meo cinnamate ligand *via* the H1*W*) atom, the other water H atom (H2*W*) being involved in the O1*W*—H2*W*⋯O_nit_ hydrogen bond (nit = nitrate anion) linking the nitrate anion to the cationic complex mol­ecule (Fig. 2 ▸). The complex moieties are inter­connected *via* moderate C—H⋯O hydrogen bonds between the 1,10-phenanthroline unit and the coordinating O atom of the 3-meo cinnamate ligand of a neighbouring complex on one side and between the 1,10-phenanthroline mol­ecules and the O atoms of the nitrate anions on the other side, generating supra­molecular hydrogen-bonded chains along the *c*-axis direction (Fig. 2 ▸). The chains are linked through slipped π–π stacking inter­actions with inter­centroid distances ranging from 3.729 (2) to 3.891 (2) Å, the most significant being between the pyridyl rings containing phenanthroline atom N4 of each mol­ecule [*Cg*4⋯*Cg*4(1 − *x*, −*y*, 1 − *z*) = 3.7998 (18) Å], forming layers parallel to the *bc* plane (Fig. 3 ▸, Table 3 ▸).

## 4. (Hirshfeld surface analysis

**Figure 4:**
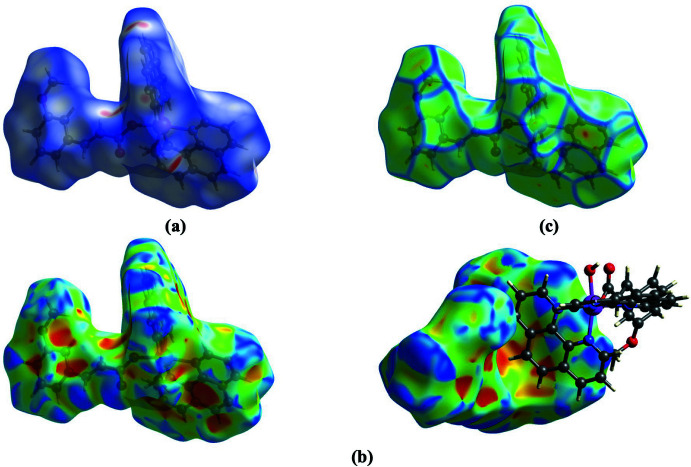
A view of the Hirshfeld surface mapped over (*a*) *d*_norm_ in the range −0.5087 to +1.3878 arbitrary units, (*b*) shape-index and (*c*) curvedness.

To further characterize the inter­molecular inter­actions in the title compound, we carried out a Hirshfeld surface (HS) analysis (Spackman & Jayatilaka, 2009 ▸) using *Crystal Explorer 21* (Spackman *et al.*, 2021 ▸) and generated the associated two-dimensional fingerprint plots (McKinnon *et al.*, 2007 ▸). The HS mapped over *d*_norm_ in the range 0.5087 to +1.3878 a.u. is illustrated in Fig. 4 ▸ using colours to indicate contacts that are shorter (red areas), equal to (white areas), or longer than (blue areas) the sum of the van der Waals radii (Ashfaq *et al.*, 2021 ▸). The red spots on the surface mapped over *d*_norm_ (Fig. 4 ▸ *a*) indicate the involvement of atoms in hydrogen-bonding inter­actions. The HS mapped over shape-index (Fig. 4 ▸ *b*) is used to check for the presence of inter­actions such as C—H⋯π and π–π stacking (Ashfaq *et al.*, 2021 ▸). The existence of adjacent red and blue triangular regions around the aromatic rings confirms the presence of π–π stacking inter­actions in the title compound (Fig. 4 ▸ *b*), and the curvedness plots (Fig. 4 ▸ *c*) show flat surface patches characteristic of planar stacking.

**Figure 5:**
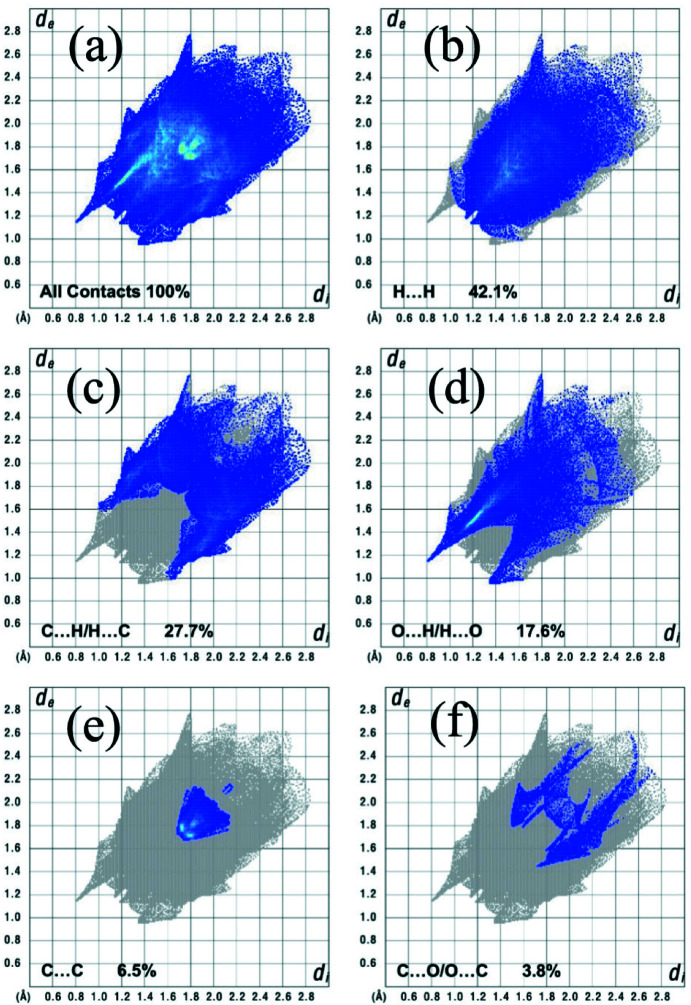
Two-dimensional fingerprint plots for the title compound, showing (*a*) all inter­actions, and delineated into (*b*) H⋯H, (*c*) C⋯H/H⋯C, (*d*) O⋯H/H⋯O, (*e*) C⋯C and (*f*) C⋯O/O⋯C inter­actions. The *d*_i_ and *d*_e_ values are the closest inter­nal and external distances (in Å) from given points on the Hirshfeld surface.

The two-dimensional fingerprint plots provide quantitative information about the non-covalent inter­actions and the crystal packing in terms of the percentage contribution of the inter­atomic contacts (Spackman & McKinnon, 2002 ▸; Ashfaq *et al.*, 2021 ▸). Fig. 5 ▸ shows the two-dimensional fingerprint plot for the overall inter­actions in the title compound with relative contributions to the Hirshfeld surface. The most important inter­atomic contact is H⋯H as it makes the highest contribution to the crystal packing (42.1%, Fig. 5 ▸ *b*). Other major contributors are C⋯H (27.7%, Fig. 5 ▸ *c*) and O⋯H (17.7%, Fig. 5 ▸ *d*) inter­actions. Smaller contributions are made by C⋯C (6.5%, Fig. 5 ▸ *e*) and C⋯O (3.8%, Fig. 5 ▸ *f*) inter­actions. Other contacts make a contribution of 2.3% in total and are not discussed in this work.

## 5. Database survey

A survey of the Cambridge Structural Database (CSD, version 5.43; update of June 2022; Groom *et al.*, 2016 ▸) revealed that crystal structures had been reported for complexes of 3-meth­oxy cinnamic acid derivatives and a number of metal ions, including copper (Drew *et al.*, 1994 ▸), cadmium (Zhang *et al.*, 2013 ▸), tin (Su *et al.*, 2022 ▸), cerium, neodymium, europium, gadolinium (Khalfaoui *et al.*, 2017 ▸, 2021 ▸) and dysprosium (Khalfaoui *et al.*, 2018 ▸, 2017 ▸). Only one complex based on copper and 2,5-di­meth­oxy­cinnamic acid with 2,9-dimethyl-1,l0-phenanthroline has been reported (Battaglia *et al.*, 1991 ▸). However, no complexes containing only the cobalt ion and 3-meth­oxy cinnamic acid associated with 1,10-phenanthroline have been documented in the CSD.

## 6. Synthesis and crystallization

A mixture of Co(NO_3_)_2_·6H_2_O (0.240 g, 1 mmol), 3-meth­oxy cinnamic acid (0.178 g, 1 mmol), NaOH (0.04 g, 1 mmol) and 1,10-phen (0.180 g, 1 mmol) were dissolved in 10 mL of mixed solution (MeOH/H_2_O: 2/1) in a 20 mL Teflon-lined stainless steel reactor and heated to 393 K for 24 h. It was then allowed to cool to room temperature in a water bath. Green crystals suitable for X-ray analysis were obtained.

## 7. Refinement

Crystal data, data collection and structure refinement details are summarized in Table 4 ▸. Hydrogen atoms of the water mol­ecule were localized in difference-Fourier maps and refined with O—H = 0.85 ±0.01 Å, and with *U*_iso_(H) set to 1.5*U*_eq_(O). The C-bound H atoms were placed in calculated positions with C—H = 0.93 or 0.96 Å and refined using a riding model with fixed isotropic displacement parameters [*U*_iso_(H) = 1.2–1.5*U*_eq_(C)]. The 3-meth­oxy cinnamate mol­ecule shows disorder over two positions with final occupancies of 0.735 (6) and 0.265 (6). The disordered atoms were modelled as anisotropic using EXYZ and EADP constraints.

## Supplementary Material

Crystal structure: contains datablock(s) I. DOI: 10.1107/S2056989022009781/jy2023sup1.cif

Structure factors: contains datablock(s) I. DOI: 10.1107/S2056989022009781/jy2023Isup4.hkl

CCDC reference: 2211433

Additional supporting information:  crystallographic information; 3D view; checkCIF report

## Figures and Tables

**Table 1:** Selected geometric parameters (Å, °)

Co1—O1*W*	2.1011 (17)	O3—C6_2	1.202 (19)
Co1—N1	2.1484 (18)	O3—C10_2	1.56 (3)
Co1—N2	2.1488 (17)	O1_1—C1_1	1.252 (5)
Co1—N3	2.1356 (16)	O2_1—C1_1	1.229 (6)
Co1—N4	2.1416 (17)	C1_1—C2_1	1.485 (6)
Co1—O1_1	2.0525 (13)	O1_2—C1_2	1.344 (13)
Co1—O1_2	2.0525 (13)	O2_2—C1_2	1.257 (14)
O3—C6_1	1.409 (6)	C1_2—C2_2	1.511 (13)
O3—C10_1	1.378 (10)	C2_2—C3_2	1.281 (13)
			
O1*W*—Co1—N1	166.30 (7)	O1_1—Co1—O1*W*	89.41 (7)
O1*W*—Co1—N2	90.34 (7)	O1_1—Co1—N1	85.52 (6)
O1*W*—Co1—N3	89.59 (7)	O1_1—Co1—N2	91.21 (6)
O1*W*—Co1—N4	95.01 (7)	O1_1—Co1—N3	169.27 (6)
N1—Co1—N2	77.08 (7)	O1_1—Co1—N4	91.71 (6)
N3—Co1—N1	97.72 (7)	O1_2—Co1—O1*W*	89.41 (7)
N3—Co1—N2	99.48 (6)	O1_2—Co1—N1	85.52 (6)
N3—Co1—N4	77.74 (6)	O1_2—Co1—N2	91.21 (6)
N4—Co1—N1	97.85 (7)	O1_2—Co1—N3	169.27 (6)
N4—Co1—N2	173.94 (6)	O1_2—Co1—N4	91.71 (6)

**Table 2:** Hydrogen-bond geometry (Å, °)

*D*—H⋯*A*	*D*—H	H⋯*A*	*D*⋯*A*	*D*—H⋯*A*
O1*W*—H1*WA*⋯O2_1	0.86	1.99	2.743 (4)	146
O1*W*—H1*WA*⋯O2_2	0.86	1.50	2.30 (2)	152
O1*W*—H1*WB*⋯O4^i^	0.86	2.55	3.093 (3)	123
O1*W*—H1*WB*⋯O5^i^	0.86	2.06	2.882 (3)	162
C13—H13⋯O6^ii^	0.93	2.34	3.123 (4)	141
C16—H16⋯O1_1^iii^	0.93	2.44	3.294 (6)	153
C16—H16⋯O1_2^iii^	0.93	2.48	3.32 (3)	151
C19—H19⋯O4^iv^	0.93	2.60	3.508 (5)	167

Symmetry codes: (i) 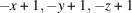 ; (ii) 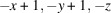 ; (iii) 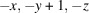 ; (iv) 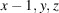 .

**Table 3:** π–π stacking inter­actions (Å)

*Cg*	Ring	*Cg*⋯*Cg*	Distance
*Cg*1	N1/C11–C14/C21	*Cg*1⋯*Cg*3^i^	3.741 (2)
*Cg*2	N2/C17–C20/C22	*Cg*2⋯*Cg*3^ii^	3.891 (2)
*Cg*3	C14–C17/C21/C22	*Cg*3⋯*Cg*3^ii^	3.729 (2)
*Cg*4	N4/C29–C32/C34	*Cg*4⋯*Cg*4^iii^	3.7998 (18)

Symmetry codes: (i) 1 − *x*, 1 − *y*, 2 − *z*; (ii) 2 − *x*, 1 − *y*, 2 − *z*; (iii) 1 − *x*, −*y*, 1 − *z*.

**Table 4:** Experimental details

Crystal data	
Chemical formula	[Co(C_10_H_9_O_3_)(C_12_H_8_N_2_)_2_(H_2_O)]NO_3_
*M*_r_	676.53
Crystal system, space group	Triclinic, *P* 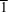
Temperature (K)	296
*a*, *b*, *c* (Å)	8.3354 (1), 13.6529 (2), 13.8423 (2)
α, β, γ (°)	101.634 (1), 98.239 (1), 97.819 (1)
*V* (Å^3^)	1504.73 (4)
*Z*	2
Radiation type	Mo *K*α
μ (mm^−1^)	0.63
Crystal size (mm)	0.2 × 0.15 × 0.12

Data collection	
Diffractometer	Bruker APEXII CCD
Absorption correction	Multi-scan (*SADABS*; Krause *et al.*, 2015 ▸)
*T*_min_, *T*_max_	0.710, 0.746
No. of measured, independent and observed [*I* > 2σ(*I*)] reflections	19640, 7385, 5301
*R*_int_	0.028
(sin θ/λ)_max_ (Å^−1^)	0.667

Refinement	
*R*[*F* ^2^ > 2σ(*F* ^2^)], *wR*(*F* ^2^), *S*	0.044, 0.128, 0.99
No. of reflections	7385
No. of parameters	527
No. of restraints	205
H-atom treatment	H-atom parameters constrained
Δρ_max_, Δρ_min_ (e Å^−3^)	0.36, −0.28

Computer programs: *APEX2* and *SAINT* (Bruker, 2013 ▸), *SHELXL2018/3* (Sheldrick, 2015 ▸), *Mercury* (Macrae *et al.*, 2020 ▸) and *OLEX2* (Dolomanov *et al.*, 2009 ▸).

## supplementary crystallographic information

### Crystal data

**Table:**

[Co(C_10_H_9_O_3_)(C_12_H_8_N_2_)_2_(H_2_O)]NO_3_	*Z* = 2
*M**_r_* = 676.53	*F*(000) = 698
Triclinic, *P*1	*D*_x_ = 1.493 Mg m^−^^3^
*a* = 8.3354 (1) Å	Mo *K*α radiation, λ = 0.71073 Å
*b* = 13.6529 (2) Å	Cell parameters from 6690 reflections
*c* = 13.8423 (2) Å	θ = 2.4–26.7°
α = 101.634 (1)°	µ = 0.63 mm^−^^1^
β = 98.239 (1)°	*T* = 296 K
γ = 97.819 (1)°	Block, green
*V* = 1504.73 (4) Å^3^	0.2 × 0.15 × 0.12 mm

### Data collection

**Table:**

Bruker APEXII CCD diffractometer	5301 reflections with *I* > 2σ(*I*)
φ and ω scans	*R*_int_ = 0.028
Absorption correction: multi-scan (SADABS; Krause *et al.*, 2015)	θ_max_ = 28.3°, θ_min_ = 3.6°
*T*_min_ = 0.710, *T*_max_ = 0.746	*h* = −11→11
19640 measured reflections	*k* = −18→18
7385 independent reflections	*l* = −17→18

### Refinement

**Table:**

Refinement on *F*^2^	Primary atom site location: iterative
Least-squares matrix: full	Hydrogen site location: mixed
*R*[*F*^2^ > 2σ(*F*^2^)] = 0.044	H-atom parameters constrained
*wR*(*F*^2^) = 0.128	*w* = 1/[σ^2^(*F*_o_^2^) + (0.077*P*)^2^] where *P* = (*F*_o_^2^ + 2*F*_c_^2^)/3
*S* = 0.99	(Δ/σ)_max_ = 0.001
7385 reflections	Δρ_max_ = 0.36 e Å^−^^3^
527 parameters	Δρ_min_ = −0.28 e Å^−^^3^
205 restraints	

### Special details

**Table:**

Geometry. All esds (except the esd in the dihedral angle between two l.s. planes) are estimated using the full covariance matrix. The cell esds are taken into account individually in the estimation of esds in distances, angles and torsion angles; correlations between esds in cell parameters are only used when they are defined by crystal symmetry. An approximate (isotropic) treatment of cell esds is used for estimating esds involving l.s. planes.

### Fractional atomic coordinates and isotropic or equivalent isotropic displacement parameters (Å^2^)

**Table:**

	*x*	*y*	*z*	*U*_iso_*/*U*_eq_	Occ. (<1)
Co1	0.65552 (3)	0.31049 (2)	0.71066 (2)	0.03697 (11)	
O3	1.2849 (2)	0.03641 (16)	1.14993 (16)	0.0728 (5)	
O1W	0.7725 (2)	0.30532 (13)	0.58544 (12)	0.0592 (4)	
H1WA	0.835428	0.260837	0.583927	0.089*	
H1WB	0.701199	0.283337	0.531887	0.089*	
N1	0.5861 (2)	0.34285 (13)	0.85588 (13)	0.0395 (4)	
N2	0.7868 (2)	0.46154 (12)	0.77585 (13)	0.0404 (4)	
N3	0.4433 (2)	0.34579 (13)	0.62737 (14)	0.0441 (4)	
N4	0.5046 (2)	0.16423 (12)	0.65426 (13)	0.0399 (4)	
C11	0.4906 (3)	0.28302 (19)	0.8960 (2)	0.0548 (6)	
H11	0.424469	0.225251	0.854461	0.066*	
C12	0.4848 (4)	0.3029 (2)	0.9986 (2)	0.0668 (8)	
H12	0.416559	0.258239	1.023817	0.080*	
C13	0.5762 (4)	0.3854 (2)	1.0603 (2)	0.0676 (8)	
H13	0.572265	0.398436	1.128404	0.081*	
C14	0.6786 (3)	0.45249 (18)	1.02188 (17)	0.0513 (6)	
C15	0.7823 (4)	0.5427 (2)	1.0813 (2)	0.0690 (8)	
H15	0.783674	0.559246	1.149995	0.083*	
C16	0.8774 (4)	0.6039 (2)	1.0403 (2)	0.0707 (9)	
H16	0.942880	0.662327	1.081130	0.085*	
C17	0.8811 (3)	0.58178 (16)	0.9352 (2)	0.0531 (6)	
C18	0.9789 (3)	0.64185 (18)	0.8874 (3)	0.0686 (8)	
H18	1.042558	0.702893	0.923961	0.082*	
C19	0.9811 (3)	0.6117 (2)	0.7893 (3)	0.0716 (8)	
H19	1.046366	0.651427	0.757723	0.086*	
C20	0.8851 (3)	0.52090 (18)	0.7354 (2)	0.0579 (6)	
H20	0.889607	0.500365	0.667730	0.070*	
C21	0.6792 (2)	0.42749 (15)	0.91820 (15)	0.0392 (5)	
C22	0.7835 (2)	0.49190 (14)	0.87450 (16)	0.0395 (5)	
C23	0.4103 (3)	0.43484 (18)	0.6157 (2)	0.0609 (7)	
H23	0.481135	0.492909	0.653155	0.073*	
C24	0.2740 (4)	0.4463 (2)	0.5498 (2)	0.0742 (9)	
H24	0.254763	0.510758	0.544739	0.089*	
C25	0.1699 (3)	0.3628 (2)	0.4932 (2)	0.0656 (7)	
H25	0.078529	0.369687	0.449515	0.079*	
C26	0.2012 (3)	0.26581 (17)	0.50107 (17)	0.0474 (5)	
C27	0.1015 (3)	0.1734 (2)	0.44251 (18)	0.0555 (6)	
H27	0.012285	0.176044	0.394954	0.067*	
C28	0.1349 (3)	0.08284 (19)	0.45521 (18)	0.0540 (6)	
H28	0.069041	0.023818	0.415812	0.065*	
C29	0.2701 (3)	0.07560 (16)	0.52828 (16)	0.0423 (5)	
C30	0.3060 (3)	−0.01706 (16)	0.54857 (19)	0.0506 (6)	
H30	0.241610	−0.078151	0.512748	0.061*	
C31	0.4351 (3)	−0.01651 (16)	0.62063 (19)	0.0523 (6)	
H31	0.457670	−0.077092	0.635824	0.063*	
C32	0.5336 (3)	0.07515 (15)	0.67175 (17)	0.0479 (5)	
H32	0.623143	0.074236	0.719928	0.058*	
C33	0.3384 (2)	0.26158 (15)	0.57085 (16)	0.0399 (5)	
C34	0.3733 (2)	0.16419 (15)	0.58484 (15)	0.0380 (4)	
O4	0.7744 (3)	0.2759 (2)	0.3583 (2)	0.1228 (10)	
O5	0.5477 (3)	0.2821 (2)	0.40039 (18)	0.1124 (9)	
O6	0.5855 (4)	0.3196 (2)	0.26417 (18)	0.1145 (9)	
N5	0.6339 (3)	0.29278 (15)	0.33897 (16)	0.0600 (5)	
O1_1	0.84054 (18)	0.24890 (11)	0.77807 (11)	0.0478 (4)	0.735 (6)
O2_1	0.9654 (7)	0.1792 (3)	0.6607 (3)	0.0608 (10)	0.735 (6)
C1_1	0.9379 (13)	0.1928 (9)	0.7468 (6)	0.0434 (12)	0.735 (6)
C2_1	1.0126 (5)	0.1398 (3)	0.8211 (3)	0.0390 (9)	0.735 (6)
H2_1	0.981835	0.150243	0.883821	0.047*	0.735 (6)
C3_1	1.1205 (4)	0.0787 (2)	0.8031 (2)	0.0407 (9)	0.735 (6)
H3_1	1.153482	0.069490	0.740960	0.049*	0.735 (6)
C4_1	1.1908 (5)	0.0247 (3)	0.8757 (3)	0.0398 (9)	0.735 (6)
C5_1	1.2001 (8)	0.0580 (4)	0.9773 (4)	0.0428 (11)	0.735 (6)
H5_1	1.163475	0.118108	1.001304	0.051*	0.735 (6)
C6_1	1.2628 (19)	0.0042 (8)	1.0448 (5)	0.0481 (15)	0.735 (6)
C7_1	1.3155 (14)	−0.0869 (6)	1.0095 (5)	0.0598 (15)	0.735 (6)
H7_1	1.356693	−0.123860	1.054046	0.072*	0.735 (6)
C8_1	1.3060 (6)	−0.1216 (4)	0.9082 (4)	0.0587 (11)	0.735 (6)
H8_1	1.339090	−0.182963	0.884323	0.070*	0.735 (6)
C9_1	1.2473 (5)	−0.0659 (3)	0.8403 (3)	0.0489 (9)	0.735 (6)
H9_1	1.245626	−0.088822	0.772145	0.059*	0.735 (6)
C10_1	1.2348 (14)	0.1263 (8)	1.1877 (8)	0.076 (2)	0.735 (6)
H10A_1	1.301732	0.181360	1.171057	0.114*	0.735 (6)
H10B_1	1.245914	0.136916	1.259195	0.114*	0.735 (6)
H10C_1	1.121772	0.123351	1.159119	0.114*	0.735 (6)
O1_2	0.84054 (18)	0.24890 (11)	0.77807 (11)	0.0478 (4)	0.265 (6)
O2_2	0.966 (2)	0.2091 (11)	0.6365 (10)	0.078 (3)	0.265 (6)
C1_2	0.944 (4)	0.205 (2)	0.7236 (14)	0.054 (5)	0.265 (6)
C2_2	1.0527 (12)	0.1394 (7)	0.7673 (8)	0.052 (3)	0.265 (6)
H2_2	1.127208	0.112505	0.729946	0.062*	0.265 (6)
C3_2	1.0476 (15)	0.1187 (10)	0.8532 (8)	0.056 (3)	0.265 (6)
H3_2	0.971590	0.147523	0.887806	0.067*	0.265 (6)
C4_2	1.1423 (15)	0.0572 (9)	0.9042 (9)	0.051 (3)	0.265 (6)
C5_2	1.170 (2)	0.0793 (12)	1.0051 (9)	0.046 (3)	0.265 (6)
H5_2	1.125983	0.132319	1.038905	0.055*	0.265 (6)
C6_2	1.263 (5)	0.026 (2)	1.0604 (14)	0.056 (5)	0.265 (6)
C7_2	1.317 (4)	−0.0586 (17)	1.0098 (14)	0.070 (6)	0.265 (6)
H7_2	1.372393	−0.098231	1.045814	0.084*	0.265 (6)
C8_2	1.289 (2)	−0.0841 (11)	0.9059 (13)	0.073 (4)	0.265 (6)
H8_2	1.324889	−0.140507	0.871479	0.088*	0.265 (6)
C9_2	1.2062 (15)	−0.0227 (11)	0.8547 (8)	0.052 (3)	0.265 (6)
H9_2	1.193466	−0.035916	0.785204	0.062*	0.265 (6)
C10_2	1.249 (3)	0.136 (3)	1.214 (2)	0.086 (9)	0.265 (6)
H10A_2	1.284087	0.192176	1.184854	0.130*	0.265 (6)
H10B_2	1.307898	0.147790	1.280935	0.130*	0.265 (6)
H10C_2	1.133217	0.130458	1.214437	0.130*	0.265 (6)

### Atomic displacement parameters (Å^2^)

**Table:**

	*U*^11^	*U*^22^	*U*^33^	*U*^12^	*U*^13^	*U*^23^
Co1	0.04017 (18)	0.03443 (16)	0.03103 (17)	0.00215 (11)	−0.00248 (12)	0.00417 (11)
O3	0.0776 (13)	0.0878 (14)	0.0628 (13)	0.0218 (11)	0.0124 (10)	0.0340 (11)
O1W	0.0683 (11)	0.0677 (11)	0.0377 (9)	0.0075 (9)	0.0080 (8)	0.0066 (8)
N1	0.0388 (9)	0.0400 (9)	0.0390 (10)	0.0053 (7)	0.0055 (8)	0.0092 (8)
N2	0.0434 (10)	0.0363 (9)	0.0377 (10)	0.0007 (7)	0.0015 (8)	0.0077 (7)
N3	0.0465 (10)	0.0401 (9)	0.0403 (10)	0.0037 (8)	−0.0035 (8)	0.0068 (8)
N4	0.0428 (9)	0.0359 (9)	0.0367 (10)	0.0026 (7)	0.0006 (8)	0.0052 (7)
C11	0.0518 (14)	0.0562 (14)	0.0625 (16)	0.0088 (11)	0.0172 (12)	0.0230 (12)
C12	0.0686 (17)	0.0821 (19)	0.072 (2)	0.0291 (15)	0.0356 (16)	0.0423 (17)
C13	0.086 (2)	0.094 (2)	0.0412 (14)	0.0505 (18)	0.0217 (14)	0.0274 (15)
C14	0.0595 (14)	0.0637 (14)	0.0337 (12)	0.0328 (12)	0.0026 (11)	0.0065 (11)
C15	0.088 (2)	0.0739 (18)	0.0364 (14)	0.0410 (16)	−0.0112 (14)	−0.0089 (13)
C16	0.0776 (19)	0.0534 (15)	0.0586 (18)	0.0238 (14)	−0.0264 (15)	−0.0208 (13)
C17	0.0455 (13)	0.0368 (11)	0.0630 (16)	0.0093 (9)	−0.0126 (11)	−0.0069 (10)
C18	0.0519 (15)	0.0341 (12)	0.103 (3)	−0.0050 (10)	−0.0111 (15)	0.0021 (14)
C19	0.0602 (16)	0.0489 (15)	0.102 (3)	−0.0084 (12)	0.0070 (16)	0.0266 (16)
C20	0.0619 (15)	0.0507 (13)	0.0598 (16)	−0.0041 (11)	0.0105 (13)	0.0187 (12)
C21	0.0412 (11)	0.0419 (11)	0.0328 (11)	0.0153 (9)	−0.0005 (9)	0.0041 (9)
C22	0.0379 (11)	0.0328 (10)	0.0407 (12)	0.0075 (8)	−0.0055 (9)	−0.0006 (8)
C23	0.0665 (16)	0.0387 (12)	0.0687 (18)	0.0071 (11)	−0.0112 (13)	0.0095 (11)
C24	0.0768 (19)	0.0530 (15)	0.088 (2)	0.0171 (14)	−0.0153 (16)	0.0214 (15)
C25	0.0567 (15)	0.0666 (16)	0.0705 (18)	0.0125 (13)	−0.0138 (13)	0.0245 (14)
C26	0.0411 (12)	0.0569 (13)	0.0409 (13)	0.0045 (10)	0.0007 (10)	0.0104 (10)
C27	0.0436 (13)	0.0727 (16)	0.0409 (13)	−0.0014 (11)	−0.0075 (10)	0.0093 (12)
C28	0.0444 (13)	0.0595 (14)	0.0432 (14)	−0.0092 (10)	−0.0008 (10)	−0.0043 (11)
C29	0.0393 (11)	0.0441 (11)	0.0349 (11)	−0.0053 (9)	0.0077 (9)	−0.0036 (9)
C30	0.0529 (14)	0.0351 (11)	0.0568 (15)	−0.0061 (9)	0.0169 (12)	−0.0021 (10)
C31	0.0589 (15)	0.0341 (11)	0.0625 (16)	0.0037 (10)	0.0137 (12)	0.0088 (10)
C32	0.0534 (13)	0.0405 (11)	0.0479 (14)	0.0053 (10)	0.0029 (11)	0.0114 (10)
C33	0.0399 (11)	0.0413 (11)	0.0346 (11)	0.0022 (9)	0.0024 (9)	0.0055 (9)
C34	0.0366 (10)	0.0393 (10)	0.0341 (11)	0.0001 (8)	0.0050 (8)	0.0043 (8)
O4	0.0810 (17)	0.161 (3)	0.128 (2)	0.0368 (17)	0.0016 (16)	0.037 (2)
O5	0.1084 (19)	0.156 (3)	0.0656 (15)	−0.0122 (17)	0.0345 (15)	0.0190 (16)
O6	0.165 (2)	0.1119 (19)	0.0640 (15)	0.0105 (17)	−0.0126 (15)	0.0454 (14)
N5	0.0821 (16)	0.0535 (12)	0.0391 (12)	−0.0011 (11)	0.0024 (11)	0.0121 (9)
O1_1	0.0471 (8)	0.0486 (8)	0.0418 (9)	0.0106 (7)	−0.0074 (7)	0.0054 (7)
O2_1	0.0756 (19)	0.064 (2)	0.049 (2)	0.0326 (18)	0.0104 (18)	0.0135 (16)
C1_1	0.041 (2)	0.039 (3)	0.041 (3)	0.0009 (16)	−0.008 (2)	0.004 (2)
C2_1	0.042 (2)	0.0394 (17)	0.033 (2)	0.0064 (13)	0.0040 (17)	0.0039 (16)
C3_1	0.0410 (16)	0.0412 (16)	0.0366 (17)	0.0061 (12)	0.0021 (13)	0.0051 (13)
C4_1	0.036 (2)	0.037 (2)	0.045 (2)	0.0068 (17)	0.0044 (17)	0.0073 (19)
C5_1	0.040 (2)	0.038 (2)	0.048 (3)	0.0077 (16)	0.003 (2)	0.006 (2)
C6_1	0.048 (3)	0.050 (4)	0.048 (2)	0.004 (3)	0.004 (2)	0.020 (2)
C7_1	0.061 (3)	0.060 (4)	0.072 (3)	0.023 (3)	0.016 (2)	0.035 (2)
C8_1	0.058 (2)	0.045 (3)	0.079 (3)	0.020 (2)	0.012 (2)	0.020 (2)
C9_1	0.046 (2)	0.044 (2)	0.057 (2)	0.0150 (15)	0.0096 (17)	0.0080 (18)
C10_1	0.093 (6)	0.080 (4)	0.047 (4)	−0.002 (3)	0.009 (3)	0.011 (3)
O1_2	0.0471 (8)	0.0486 (8)	0.0418 (9)	0.0106 (7)	−0.0074 (7)	0.0054 (7)
O2_2	0.084 (6)	0.096 (9)	0.076 (7)	0.051 (6)	0.033 (6)	0.030 (6)
C1_2	0.053 (10)	0.037 (9)	0.052 (10)	0.013 (8)	−0.028 (7)	−0.010 (8)
C2_2	0.047 (5)	0.053 (5)	0.045 (6)	0.009 (4)	−0.004 (5)	−0.007 (4)
C3_2	0.047 (6)	0.061 (7)	0.050 (7)	0.008 (5)	−0.006 (5)	0.000 (5)
C4_2	0.048 (6)	0.046 (6)	0.054 (6)	0.011 (4)	0.007 (5)	0.000 (4)
C5_2	0.053 (8)	0.038 (7)	0.042 (6)	0.005 (5)	0.001 (6)	0.002 (5)
C6_2	0.035 (8)	0.061 (12)	0.076 (9)	0.009 (9)	0.008 (8)	0.023 (7)
C7_2	0.067 (9)	0.065 (12)	0.092 (8)	0.032 (10)	0.002 (7)	0.047 (8)
C8_2	0.076 (8)	0.048 (8)	0.106 (8)	0.034 (6)	0.025 (7)	0.018 (8)
C9_2	0.055 (7)	0.059 (9)	0.047 (6)	0.023 (7)	0.016 (5)	0.011 (6)
C10_2	0.046 (9)	0.117 (16)	0.081 (19)	0.026 (9)	0.001 (9)	−0.013 (11)

### Geometric parameters (Å, º)

**Table:**

Co1—O1W	2.1011 (17)	C28—C29	1.430 (3)
Co1—N1	2.1484 (18)	C29—C30	1.411 (3)
Co1—N2	2.1488 (17)	C29—C34	1.399 (3)
Co1—N3	2.1356 (16)	C30—H30	0.9300
Co1—N4	2.1416 (17)	C30—C31	1.356 (3)
Co1—O1_1	2.0525 (13)	C31—H31	0.9300
Co1—O1_2	2.0525 (13)	C31—C32	1.392 (3)
O3—C6_1	1.409 (6)	C32—H32	0.9300
O3—C10_1	1.378 (10)	C33—C34	1.443 (3)
O3—C6_2	1.202 (19)	O4—N5	1.227 (3)
O3—C10_2	1.56 (3)	O5—N5	1.207 (3)
O1W—H1WA	0.8536	O6—N5	1.201 (3)
O1W—H1WB	0.8535	O1_1—C1_1	1.252 (5)
N1—C11	1.320 (3)	O2_1—C1_1	1.229 (6)
N1—C21	1.358 (3)	C1_1—C2_1	1.485 (6)
N2—C20	1.333 (3)	C2_1—H2_1	0.9300
N2—C22	1.350 (3)	C2_1—C3_1	1.324 (4)
N3—C23	1.318 (3)	C3_1—H3_1	0.9300
N3—C33	1.366 (3)	C3_1—C4_1	1.465 (4)
N4—C32	1.334 (2)	C4_1—C5_1	1.374 (6)
N4—C34	1.349 (2)	C4_1—C9_1	1.404 (5)
C11—H11	0.9300	C5_1—H5_1	0.9300
C11—C12	1.400 (4)	C5_1—C6_1	1.389 (6)
C12—H12	0.9300	C6_1—C7_1	1.395 (6)
C12—C13	1.330 (4)	C7_1—H7_1	0.9300
C13—H13	0.9300	C7_1—C8_1	1.374 (6)
C13—C14	1.401 (4)	C8_1—H8_1	0.9300
C14—C15	1.427 (4)	C8_1—C9_1	1.398 (5)
C14—C21	1.407 (3)	C9_1—H9_1	0.9300
C15—H15	0.9300	C10_1—H10A_1	0.9600
C15—C16	1.338 (4)	C10_1—H10B_1	0.9600
C16—H16	0.9300	C10_1—H10C_1	0.9600
C16—C17	1.430 (4)	O1_2—C1_2	1.344 (13)
C17—C18	1.402 (4)	O2_2—C1_2	1.257 (14)
C17—C22	1.411 (3)	C1_2—C2_2	1.511 (13)
C18—H18	0.9300	C2_2—H2_2	0.9300
C18—C19	1.341 (4)	C2_2—C3_2	1.281 (13)
C19—H19	0.9300	C3_2—H3_2	0.9300
C19—C20	1.385 (4)	C3_2—C4_2	1.445 (12)
C20—H20	0.9300	C4_2—C5_2	1.346 (13)
C21—C22	1.433 (3)	C4_2—C9_2	1.379 (12)
C23—H23	0.9300	C5_2—H5_2	0.9300
C23—C24	1.399 (3)	C5_2—C6_2	1.390 (14)
C24—H24	0.9300	C6_2—C7_2	1.386 (15)
C24—C25	1.357 (4)	C7_2—H7_2	0.9300
C25—H25	0.9300	C7_2—C8_2	1.387 (15)
C25—C26	1.406 (3)	C8_2—H8_2	0.9300
C26—C27	1.432 (3)	C8_2—C9_2	1.393 (13)
C26—C33	1.403 (3)	C9_2—H9_2	0.9300
C27—H27	0.9300	C10_2—H10A_2	0.9600
C27—C28	1.343 (3)	C10_2—H10B_2	0.9600
C28—H28	0.9300	C10_2—H10C_2	0.9600
			
O1W—Co1—N1	166.30 (7)	C29—C28—H28	119.4
O1W—Co1—N2	90.34 (7)	C30—C29—C28	123.7 (2)
O1W—Co1—N3	89.59 (7)	C34—C29—C28	119.5 (2)
O1W—Co1—N4	95.01 (7)	C34—C29—C30	116.8 (2)
N1—Co1—N2	77.08 (7)	C29—C30—H30	120.2
N3—Co1—N1	97.72 (7)	C31—C30—C29	119.6 (2)
N3—Co1—N2	99.48 (6)	C31—C30—H30	120.2
N3—Co1—N4	77.74 (6)	C30—C31—H31	120.1
N4—Co1—N1	97.85 (7)	C30—C31—C32	119.7 (2)
N4—Co1—N2	173.94 (6)	C32—C31—H31	120.1
O1_1—Co1—O1W	89.41 (7)	N4—C32—C31	122.5 (2)
O1_1—Co1—N1	85.52 (6)	N4—C32—H32	118.7
O1_1—Co1—N2	91.21 (6)	C31—C32—H32	118.7
O1_1—Co1—N3	169.27 (6)	N3—C33—C26	123.34 (18)
O1_1—Co1—N4	91.71 (6)	N3—C33—C34	117.24 (17)
O1_2—Co1—O1W	89.41 (7)	C26—C33—C34	119.42 (19)
O1_2—Co1—N1	85.52 (6)	N4—C34—C29	123.39 (18)
O1_2—Co1—N2	91.21 (6)	N4—C34—C33	117.16 (17)
O1_2—Co1—N3	169.27 (6)	C29—C34—C33	119.44 (18)
O1_2—Co1—N4	91.71 (6)	O5—N5—O4	115.9 (3)
C10_1—O3—C6_1	117.1 (5)	O6—N5—O4	122.1 (3)
C6_2—O3—C10_2	117.8 (15)	O6—N5—O5	121.9 (3)
Co1—O1W—H1WA	109.4	C1_1—O1_1—Co1	134.6 (4)
Co1—O1W—H1WB	109.6	O1_1—C1_1—C2_1	114.6 (4)
H1WA—O1W—H1WB	104.3	O2_1—C1_1—O1_1	123.1 (4)
C11—N1—Co1	128.29 (17)	O2_1—C1_1—C2_1	122.2 (4)
C11—N1—C21	117.3 (2)	C1_1—C2_1—H2_1	118.1
C21—N1—Co1	113.07 (13)	C3_1—C2_1—C1_1	123.8 (4)
C20—N2—Co1	128.43 (17)	C3_1—C2_1—H2_1	118.1
C20—N2—C22	117.6 (2)	C2_1—C3_1—H3_1	118.2
C22—N2—Co1	113.33 (13)	C2_1—C3_1—C4_1	123.6 (4)
C23—N3—Co1	129.26 (16)	C4_1—C3_1—H3_1	118.2
C23—N3—C33	117.30 (18)	C5_1—C4_1—C3_1	122.5 (4)
C33—N3—Co1	113.02 (12)	C5_1—C4_1—C9_1	118.6 (4)
C32—N4—Co1	128.27 (14)	C9_1—C4_1—C3_1	118.8 (4)
C32—N4—C34	117.93 (18)	C4_1—C5_1—H5_1	119.1
C34—N4—Co1	113.42 (12)	C4_1—C5_1—C6_1	121.7 (4)
N1—C11—H11	118.6	C6_1—C5_1—H5_1	119.1
N1—C11—C12	122.7 (3)	C5_1—C6_1—O3	124.9 (5)
C12—C11—H11	118.6	C5_1—C6_1—C7_1	119.6 (5)
C11—C12—H12	119.8	C7_1—C6_1—O3	115.4 (5)
C13—C12—C11	120.4 (3)	C6_1—C7_1—H7_1	120.3
C13—C12—H12	119.8	C8_1—C7_1—C6_1	119.4 (5)
C12—C13—H13	120.2	C8_1—C7_1—H7_1	120.3
C12—C13—C14	119.6 (2)	C7_1—C8_1—H8_1	119.5
C14—C13—H13	120.2	C7_1—C8_1—C9_1	121.0 (4)
C13—C14—C15	124.1 (2)	C9_1—C8_1—H8_1	119.5
C13—C14—C21	117.1 (2)	C4_1—C9_1—H9_1	120.2
C21—C14—C15	118.8 (3)	C8_1—C9_1—C4_1	119.6 (4)
C14—C15—H15	119.3	C8_1—C9_1—H9_1	120.2
C16—C15—C14	121.4 (3)	O3—C10_1—H10A_1	109.5
C16—C15—H15	119.3	O3—C10_1—H10B_1	109.5
C15—C16—H16	119.2	O3—C10_1—H10C_1	109.5
C15—C16—C17	121.7 (2)	H10A_1—C10_1—H10B_1	109.5
C17—C16—H16	119.2	H10A_1—C10_1—H10C_1	109.5
C18—C17—C16	124.7 (3)	H10B_1—C10_1—H10C_1	109.5
C18—C17—C22	116.8 (2)	C1_2—O1_2—Co1	120.1 (7)
C22—C17—C16	118.5 (3)	O1_2—C1_2—C2_2	119.5 (11)
C17—C18—H18	119.8	O2_2—C1_2—O1_2	129.7 (11)
C19—C18—C17	120.3 (2)	O2_2—C1_2—C2_2	110.8 (11)
C19—C18—H18	119.8	C1_2—C2_2—H2_2	118.4
C18—C19—H19	120.3	C3_2—C2_2—C1_2	123.3 (11)
C18—C19—C20	119.4 (3)	C3_2—C2_2—H2_2	118.4
C20—C19—H19	120.3	C2_2—C3_2—H3_2	115.2
N2—C20—C19	123.2 (3)	C2_2—C3_2—C4_2	129.6 (11)
N2—C20—H20	118.4	C4_2—C3_2—H3_2	115.2
C19—C20—H20	118.4	C5_2—C4_2—C3_2	118.3 (11)
N1—C21—C14	123.0 (2)	C5_2—C4_2—C9_2	118.4 (10)
N1—C21—C22	117.13 (18)	C9_2—C4_2—C3_2	123.3 (11)
C14—C21—C22	119.9 (2)	C4_2—C5_2—H5_2	118.9
N2—C22—C17	122.7 (2)	C4_2—C5_2—C6_2	122.2 (12)
N2—C22—C21	117.57 (18)	C6_2—C5_2—H5_2	118.9
C17—C22—C21	119.7 (2)	O3—C6_2—C5_2	126.2 (16)
N3—C23—H23	118.4	O3—C6_2—C7_2	114.4 (15)
N3—C23—C24	123.2 (2)	C7_2—C6_2—C5_2	118.6 (15)
C24—C23—H23	118.4	C6_2—C7_2—H7_2	119.8
C23—C24—H24	120.2	C6_2—C7_2—C8_2	120.4 (13)
C25—C24—C23	119.6 (2)	C8_2—C7_2—H7_2	119.8
C25—C24—H24	120.2	C7_2—C8_2—H8_2	121.0
C24—C25—H25	120.2	C7_2—C8_2—C9_2	118.1 (11)
C24—C25—C26	119.5 (2)	C9_2—C8_2—H8_2	121.0
C26—C25—H25	120.2	C4_2—C9_2—C8_2	121.8 (11)
C25—C26—C27	123.7 (2)	C4_2—C9_2—H9_2	119.1
C33—C26—C25	116.9 (2)	C8_2—C9_2—H9_2	119.1
C33—C26—C27	119.4 (2)	O3—C10_2—H10A_2	109.5
C26—C27—H27	119.5	O3—C10_2—H10B_2	109.5
C28—C27—C26	121.0 (2)	O3—C10_2—H10C_2	109.5
C28—C27—H27	119.5	H10A_2—C10_2—H10B_2	109.5
C27—C28—H28	119.4	H10A_2—C10_2—H10C_2	109.5
C27—C28—C29	121.2 (2)	H10B_2—C10_2—H10C_2	109.5
			
Co1—N1—C11—C12	164.56 (16)	C24—C25—C26—C33	2.0 (4)
Co1—N1—C21—C14	−167.13 (14)	C25—C26—C27—C28	−178.2 (3)
Co1—N1—C21—C22	11.8 (2)	C25—C26—C33—N3	−2.4 (3)
Co1—N2—C20—C19	−170.90 (18)	C25—C26—C33—C34	177.8 (2)
Co1—N2—C22—C17	170.42 (14)	C26—C27—C28—C29	0.6 (4)
Co1—N2—C22—C21	−7.8 (2)	C26—C33—C34—N4	−178.66 (19)
Co1—N3—C23—C24	172.6 (2)	C26—C33—C34—C29	0.3 (3)
Co1—N3—C33—C26	−172.22 (17)	C27—C26—C33—N3	177.7 (2)
Co1—N3—C33—C34	7.6 (2)	C27—C26—C33—C34	−2.1 (3)
Co1—N4—C32—C31	−171.81 (17)	C27—C28—C29—C30	176.6 (2)
Co1—N4—C34—C29	171.32 (16)	C27—C28—C29—C34	−2.5 (4)
Co1—N4—C34—C33	−9.8 (2)	C28—C29—C30—C31	−178.7 (2)
Co1—O1_1—C1_1—O2_1	17.0 (18)	C28—C29—C34—N4	−179.1 (2)
Co1—O1_1—C1_1—C2_1	−160.3 (4)	C28—C29—C34—C33	2.0 (3)
Co1—O1_2—C1_2—O2_2	13 (5)	C29—C30—C31—C32	−1.9 (4)
Co1—O1_2—C1_2—C2_2	−165.8 (18)	C30—C29—C34—N4	1.7 (3)
O3—C6_1—C7_1—C8_1	−176.9 (10)	C30—C29—C34—C33	−177.16 (19)
O3—C6_2—C7_2—C8_2	175 (3)	C30—C31—C32—N4	1.4 (4)
N1—C11—C12—C13	0.7 (4)	C32—N4—C34—C29	−2.2 (3)
N1—C21—C22—N2	−2.8 (2)	C32—N4—C34—C33	176.65 (19)
N1—C21—C22—C17	178.98 (15)	C33—N3—C23—C24	0.6 (4)
N3—C23—C24—C25	−0.9 (5)	C33—C26—C27—C28	1.7 (4)
N3—C33—C34—N4	1.5 (3)	C34—N4—C32—C31	0.6 (3)
N3—C33—C34—C29	−179.61 (19)	C34—C29—C30—C31	0.4 (3)
C11—N1—C21—C14	0.7 (3)	O1_1—C1_1—C2_1—C3_1	−178.8 (7)
C11—N1—C21—C22	179.60 (17)	O2_1—C1_1—C2_1—C3_1	3.8 (15)
C11—C12—C13—C14	0.1 (4)	C1_1—C2_1—C3_1—C4_1	−178.4 (7)
C12—C13—C14—C15	−179.6 (2)	C2_1—C3_1—C4_1—C5_1	−24.1 (6)
C12—C13—C14—C21	−0.5 (3)	C2_1—C3_1—C4_1—C9_1	154.3 (4)
C13—C14—C15—C16	−180.0 (2)	C3_1—C4_1—C5_1—C6_1	178.1 (9)
C13—C14—C21—N1	0.1 (3)	C3_1—C4_1—C9_1—C8_1	−176.3 (4)
C13—C14—C21—C22	−178.81 (18)	C4_1—C5_1—C6_1—O3	176.2 (10)
C14—C15—C16—C17	−0.5 (4)	C4_1—C5_1—C6_1—C7_1	−1.1 (18)
C14—C21—C22—N2	176.21 (16)	C5_1—C4_1—C9_1—C8_1	2.1 (7)
C14—C21—C22—C17	−2.0 (3)	C5_1—C6_1—C7_1—C8_1	1 (2)
C15—C14—C21—N1	179.32 (17)	C6_1—C7_1—C8_1—C9_1	1.2 (15)
C15—C14—C21—C22	0.4 (3)	C7_1—C8_1—C9_1—C4_1	−2.6 (9)
C15—C16—C17—C18	−179.3 (2)	C9_1—C4_1—C5_1—C6_1	−0.3 (11)
C15—C16—C17—C22	−1.2 (3)	C10_1—O3—C6_1—C5_1	2.4 (18)
C16—C17—C18—C19	176.1 (2)	C10_1—O3—C6_1—C7_1	179.8 (11)
C16—C17—C22—N2	−175.75 (18)	O1_2—C1_2—C2_2—C3_2	3 (4)
C16—C17—C22—C21	2.4 (3)	O2_2—C1_2—C2_2—C3_2	−176.3 (19)
C17—C18—C19—C20	0.3 (4)	C1_2—C2_2—C3_2—C4_2	180 (2)
C18—C17—C22—N2	2.5 (3)	C2_2—C3_2—C4_2—C5_2	151.7 (16)
C18—C17—C22—C21	−179.31 (18)	C2_2—C3_2—C4_2—C9_2	−28 (2)
C18—C19—C20—N2	1.3 (4)	C3_2—C4_2—C5_2—C6_2	−179 (3)
C20—N2—C22—C17	−1.1 (3)	C3_2—C4_2—C9_2—C8_2	−176.4 (13)
C20—N2—C22—C21	−179.30 (18)	C4_2—C5_2—C6_2—O3	−175 (3)
C21—N1—C11—C12	−1.1 (3)	C4_2—C5_2—C6_2—C7_2	−5 (5)
C21—C14—C15—C16	0.9 (3)	C5_2—C4_2—C9_2—C8_2	4 (2)
C22—N2—C20—C19	−0.9 (3)	C5_2—C6_2—C7_2—C8_2	5 (6)
C22—C17—C18—C19	−2.1 (3)	C6_2—C7_2—C8_2—C9_2	0 (4)
C23—N3—C33—C26	1.1 (3)	C7_2—C8_2—C9_2—C4_2	−5 (3)
C23—N3—C33—C34	−179.1 (2)	C9_2—C4_2—C5_2—C6_2	1 (3)
C23—C24—C25—C26	−0.5 (5)	C10_2—O3—C6_2—C5_2	−18 (6)
C24—C25—C26—C27	−178.1 (3)	C10_2—O3—C6_2—C7_2	172 (3)

### Hydrogen-bond geometry (Å, º)

**Table:**

*D*—H···*A*	*D*—H	H···*A*	*D*···*A*	*D*—H···*A*
O1*W*—H1*WA*···O2_1	0.86	1.99	2.743 (4)	146
O1*W*—H1*WA*···O2_2	0.86	1.50	2.30 (2)	152
O1*W*—H1*WB*···O4^i^	0.86	2.55	3.093 (3)	123
O1*W*—H1*WB*···O5^i^	0.86	2.06	2.882 (3)	162
C13—H13···O6^ii^	0.93	2.34	3.123 (4)	141
C16—H16···O1_1^iii^	0.93	2.44	3.294 (6)	153
C16—H16···O1_2^iii^	0.93	2.48	3.32 (3)	151
C19—H19···O4^iv^	0.93	2.60	3.508 (5)	167

Symmetry codes: (i) −*x*+1, −*y*+1, −*z*+1; (ii) −*x*+1, −*y*+1, −*z*; (iii) −*x*, −*y*+1, −*z*; (iv) *x*−1, *y*, *z*.
